# Demographics and Outcomes of Patients With Eating Disorders Treated in Residential Care

**DOI:** 10.3389/fpsyg.2019.02985

**Published:** 2020-01-17

**Authors:** Martin Fisher, Jennifer R. Henretty, Shelbi A. Cox, Ronald Feinstein, Victor Fornari, Lindsay Moskowitz, Marcie Schneider, Sara Levine, Joan Malizio, Joanna Fishbein

**Affiliations:** ^1^Division of Adolescent Medicine, Cohen Children’s Medical Center, Northwell Health, New Hyde Park, NY, United States; ^2^Hofstra-Northwell School of Medicine, Hempstead, NY, United States; ^3^Department of Outcomes and Research, Center For Discovery, Los Alamitos, CA, United States; ^4^Division of Child and Adolescent Psychiatry, Zucker Hillside Hospital, Northwell Health, New Hyde Park, NY, United States; ^5^Greenwich Adolescent Medicine, Greenwich, CT, United States; ^6^Biostatistics Unit, Feinstein Institute for Medical Research, Northwell Health, Manhasset, NY, United States

**Keywords:** eating disorder, anorexia, bulimia, residential treatment, outcome, adolescent, adult

## Abstract

The use of residential eating disorder (ED) treatment has grown dramatically in the United States, yet there has been minimal evaluation of treatment outcomes. Thus, outcome data on weight restoration, purging behaviors, and/or Global Assessment of Functioning (GAF) for 1,421 patients treated over an 8-year period in residential ED programs are described. Results suggest that, (1) for patients who needed weight restoration upon admission, adolescent and adult patients gained 2.0 and 2.1 lb/week, respectively; (2) of patients who reported purge behavior the month before admission, 89.1% were able to completely cease purging while in treatment; (3) although improvement of approximately 10 mean GAF points was made during treatment, patients were still quite impaired at discharge; and (4) mean length of stay was 12 days longer for adolescents than adults, and 10–15 days longer for patients diagnosed with anorexia compared to bulimia or ED Not Otherwise Specified (EDNOS), respectively. Other demographic statistics and additional analyses are presented. Limitations include the high variance of purging data and reliance on self- and parent-report for admission data. The data on the 1,421 patients, which represents 96% of all patients treated during the study period, more than doubles the number of residential ED patients with outcome in the literature.

## Introduction

With decreases in insurance reimbursement in the 1990s for inpatient hospital treatment for patients with eating disorders (EDs), alternative levels-of-care, including that of residential treatment, were developed for management of the medical, nutritional, and mental health care needs of the ED patient population in the United States. Today in the United States, although around-the-clock-care is provided at both residential ED treatment and inpatient hospitalization, residential care provides primarily psychological treatment (with a medical component) to medically-stabilized patients, whereas inpatient hospitalization usually provides only short-term medical stabilization, often without ED-specific psychological treatment and often at a significantly greater daily cost than residential treatment. However, in the 30 years since its inception, residential ED treatment has received relatively little study. To date, there have been a total of eight studies examining admission-to-discharge outcomes in residential ED treatment. A review by Friedman et al. (2016) summarized the results of six of the studies (i.e., Lowe et al., 2003; McHugh, 2007; Delinsky et al., 2010; Hoffart et al., 2010; Brewerton and Costin, 2011; Weltzin et al., 2014); a study by Bean et al. (2004), published in 2004, was not included. Since the review, Twohig et al. (2016) have published one additional study. These eight studies, which had sample sizes between 33 and 470 patients, represented 66% of the patients treated during the study period in the residential programs that were studied. Results showed weight gain in the patients with anorexia nervosa, decreased bingeing/purging in the patients with bulimia nervosa (BN), and psychological improvements in patients generally.

While there is a dearth of outcome data, residential ED treatment centers are increasing. In a 10-year period, the number of ED residential treatment centers in the United States grew from 22 in 2006 (Frisch et al., 2006) to at least 75 as of 2014 (Twohig et al., 2016). Thus, it may be reasonable to surmise that fewer than 10% of residential ED programs have had their outcomes examined. Understandably, in 2016, a paper by Attia et al. (2016) entitled, “Marketing Residential Treatment Programs for Eating Disorders: A Call for Transparency,” made a plea for an increase in the number of outcome studies being performed in ED residential programs. Especially given that approximately 30% of ED patients remain ill 10–20 years following treatment (Keel and Brown, 2010), with readmission rates for inpatient hospitalization cited to be as high as 45.0–77.5% in the literature (Lay et al., 2002; Steinhausen et al., 2008), more outcome data are critical.

This study corresponds to that need and describes outcome data for over 1,400 patients treated for EDs in 12 residential facilities operated by one large treatment program in the United States over an 8-year period. The data were analyzed and are being presented by the Divisions of Adolescent Medicine and Child and Adolescent Psychiatry, along with the Biostatistics Unit, of Northwell Health, an academic medical center in New York. The data demonstrate differences between admission and discharge for patients with the DSM-IV-TR (American Psychiatric Association, 2000) diagnoses of anorexia nervosa restricting type (AN-R), anorexia nervosa purging type (AN-P), BN, and ED not otherwise specified (EDNOS).

## Materials and Methods

### Treatment Program

Center For Discovery, which opened in 1997, operates residential ED treatment facilities, each with a maximum census of six or eight patients. Patients who are admitted to a Center For Discovery residential facility live at the treatment facility, generally for more than 1 month. Some of the (gender-inclusive) facilities treat only adolescent patients, aged 10–19, while others treat only female-identifying adults. Between 2006 and 2013, Center For Discovery operated as many as 12 residential facilities in California, Washington, Illinois, and Connecticut.

Center For Discovery is like other ED residential treatment in many ways. Weekly programming includes two to three individual sessions; one to two family sessions; dietary, medical, and psychiatric sessions; and between 35 and 40 therapeutic groups. Modalities such as Exposure Response Prevention, Dialectical Behavioral Therapy, Cognitive Behavioral Therapy, Acceptance and Commitment Therapy, and a Family Systems Approach are utilized. However, according to their outreach material, there are several factors that make Center For Discovery unique: (a) Treatment takes place in actual homes in residential neighborhoods, rather than in large, institutional settings. (b) Patients with EDs are not mixed with patients with other primary mental health disorders—that is, although it is common for patients to present with co-occurring diagnoses (e.g., generalized anxiety disorder, major depressive disorder), all patients admitted into the program have a primary ED diagnosis and all treatment provided is specialized for patients diagnosed with an ED. (c) Because Center For Discovery treats such small groups of patients in each, the patient to staff ratio is very high, with typically only one to three patients for every one staff during waking hours (which allows for an extremely high level of supervision) (Hoffart et al., 2010). All staff therapists and dietitians are sponsored by Center For Discovery to become Certified Eating Disorder Specialist through the International Association of Eating Disorder Professionals (Lowe et al., 2003). Because treatment takes place in homes with kitchens (rather than in institutional settings with cafeterias), treatment includes intensive “hands on” exposure, with patients planning and preparing their own meals, which makes treatment gains more generalizable on the path to intuitive eating (McHugh, 2007). The Discovery Aftercare Program provides support and resources for patients and their families after discharge to facilitate long-term recovery.

### Data Collection

Between 2006 and 2013, patient data were collected at the time of admission and discharge via standardized measures, and post-discharge via a retrospective chart review. This study utilizes the data that were collected retrospectively from paper charts, and includes age; gender; race and ethnicity; DSM-IV-TR (American Psychiatric Association, 2000) ED diagnosis; number of treatment days; prior treatment; and, for admission and discharge, weight and height, frequency of purging, and Global Assessment of Functioning (GAF) Scores.

Global Assessment of Functioning scores were established by the treating, licensed (or supervised by a licensed) therapist for each patient on admission and discharge, as described in the DSM-IV-TR (American Psychiatric Association, 2000). Diagnosis also was assigned by the therapist, per DSM-IV-TR criteria, utilizing current reports/evidence and, when appropriate, diagnoses from previous treatment. Number of purge episodes was identified via patient self-report on admission and per clinician report on discharge. Weight was measured upon admission and weekly throughout treatment; the last weight in the chart was utilized for discharge weight.

As what constitutes a “healthy” body mass index (BMI; weight in kilograms divided by height in meters squared) is not standardized for children/adolescents (i.e., whereas a BMI of 16.5 is typically considered severely “underweight” at below the fifth percentile for a 17-year-old girl, a BMI of 16.5 is considered “healthy” at the 50th percentile for a 10-year-old girl), BMI is only provided for adult patients in this study (though BMI change is noted for both adolescents and adults). In presenting the BMI data, the authors acknowledge the research indicating that BMI is often a poor indicator of health both in children and adolescents, as summarized in a review by Bacon and Aphramor (2011).

For both adult and adolescent patients, percent of minimum target body weight (%TBW) is used as a standardized marker of health in this study and was calculated per Center For Discovery protocol: For patients diagnosed with AN and treated at the adolescent facilities, historical growth charts were utilized, often targeting the 50th percentile for height and age, to identify a minimum TBW; current weight was divided by TBW to identify %TBW. For patients (all of whom identified as female) diagnosed with AN treated at the adult facilities, the HAMWI method was utilized to calculate TBW—that is, 100 pounds (lb) for 60 inches and 5 lb for every inch above 60 inches. Current weight was then divided by the TBW to identify %TBW for adult patients. Per protocol, if the treating physician or dietitian felt that this minimum TBW was too low to provide physical and psychological remission from ED symptoms, this TBW could be adjusted for both adolescent and adult patients. It should be noted that the concept of minimum TBW replaces the term ideal body weight (IBW), which has been used for many years in the ED field, because the word “ideal” in this context is both value-laden and imprecise.

Of the 1,485 patients treated (>1 day) at Center For Discovery ED residential facilities between 2006 and 2013, 1,421 (95.7%) were included in this study. Inclusion criteria were: (a) first admission to Center For Discovery, (b) data on ED diagnosis, and (c) data on patient age. As fewer of the facilities were dedicated to adults (three facilities) than were dedicated to adolescents (eight facilities), it was not surprising that the sample was predominately adolescent (68.9%). So as not to skew the descriptive data unrepresentatively, the samples were examined separately.

Of the 979 adolescent patients, 91.0% identified as female, 8.9% identified as male, and 0.1% (1 patient) were unspecified; 73.0% identified as White, 13.7% as Hispanic, 2.7% as Asian, 1.0% as Black, 5.7% as multiracial/multiethnic, 1.8% as other races/ethnicities, and 2.0% (20 patients) did not have data for race/ethnicity. Of the 442 adult patients, 98.0% identified as female and, as some of the (gender-inclusive) adolescent facilities treated patients up to age 19, 2.0% (nine patients) identified as male; 77.8% of adult patients identified as White, 11.31% as Hispanic, 1.6% as Asian, 0.9% as Black, 4.1% as multiracial/multiethnic, 0.9% as other races/ethnicities, and 3.4% (15 patients) did not have data for race/ethnicity.

### Data Analyses

In 2014, Center For Discovery sent their data for the years 2006 through 2013 to the Biostatistics Unit and the Divisions of Adolescent Medicine and Child/Adolescent Psychiatry of the North Shore-Long Island Jewish Health System (now Northwell Health) for analysis and interpretation. Descriptive statistics were calculated to explore the demographics of the sample and to determine: (a) the changes in GAF score experienced by all patients, (b) the changes in purging behaviors experienced by patients diagnosed with AN-P and BN, and (c) the changes in weight experienced by patients diagnosed with AN.

## Results

### Treatment Parameters of the Total Samples of Adolescent and Adult Patients

Of the 1,421 patients in this study, 979 were adolescents with an average age of 15 years (*SD* = 1.6) and 442 were adults with an average age of almost 25 years (*SD* = 9.2). As evident in Table 1, most patients had received prior treatment, with over 80% of adults (*n* = 337) and almost 90% of adolescents (*n* = 824) having received prior outpatient treatment and almost half of all adolescents (*n* = 496) and adults (*n* = 187) having been hospitalized prior to Center For Discovery. Fewer patients, especially adolescent patients, had received prior treatment on the middle of the continuum of care (i.e., intensive outpatient, partial hospitalization, and residential treatment). In total, 96.9% of the patients (*n* = 1,338) had at least one form of prior treatment.

**TABLE 1:** Treatment parameters of adolescent and adult ED residential patients.

	**Adolescent**			**Adult**		
		
**Parameters**	***n***	***M***	**(*SD*)**	***n***	***M***	**(*SD*)**
Age	979	15.1	(1.6)	442	24.9	(9.2)

**Prior treatment^a^**	***n***	**% of Total**		***n***	**% of Total**	

Hospitalization	496	53.4%		187	46.3%	
Residential	92	10.0%		110	27.3%	
Partial hospitalization	149	16.3%		89	22.2%	
Intensive outpatient	155	16.9%		83	21.1%	
Outpatient	824	88.1%		337	81.2%	

**Primary diagnosis**	***n***	**% of Total**		***n***	**% of Total**	

AN–R	399	40.8%		120	27.1%	
AN–P	146	14.9%		78	17.6%	
BN	306	31.3%		188	42.5%	
EDNOS	128	13.1%		56	12.7%	

**Length of stay in days**	***n***	***M***	**(*SD*)**	***n***	***M***	**(*SD*)**

AN-R	399	55.9	(30.6)	120	41.7	(25.7)
AN-P	146	50.1	(25.9)	78	42.5	(30.1)
BN	306	40.6	(25.1)	188	31.1	(20.0)
EDNOS	128	36.7	(26.5)	56	25.4	(18.0)

*^*a*^Patients often had more than one type of prior treatment, therefore here percent data do not sum to 100. Percent was taken out of total available data. Missing data for Prior Treatment were as follows: Of the 979 adolescent patients, 50 patients were missing data for prior hospitalization, 63 patients were missing data for prior (non-Center For Discovery) residential, 63 patients were missing data for partial hospitalization, 61 patients were missing for prior intensive outpatient, and 44 patients were missing data for prior outpatient treatment. Of the 442 adult patients, 38 patients were missing data for prior hospitalization, 39 patients were missing data for prior (non-Center For Discovery) residential, 41 patients were missing data for partial hospitalization, 48 patients were missing data for prior intensive outpatient, and 27 patients were missing data for prior outpatient treatment.*

Examining diagnosis, approximately half of both adolescents and adults were diagnosed with a type of AN (AN-R or AN-P), about half of both adults and adolescents were treated for purging behaviors (AN-P or BN), and a similar percent of adolescents and adults had the diagnosis of either AN-P or EDNOS (see Table 1). There were, however, differences between adolescent and adult patients: Approximately 55% of adolescent patients (*n* = 545) were diagnosed with AN, whereas approximately 45% of adult patients (*n* = 198) were diagnosed with AN. On the contrary, approximately 45% of adolescent patients (*n* = 452) were treated for purge behaviors, whereas 60% of adult patients (*n* = 266) were treated for purge behaviors. The difference between the percentage of adults and the percentage of adolescents diagnosed with BN was over 10%, with adults more frequently receiving the BN diagnosis. Of importance to note, with the edition of DSM-5 (American Psychiatric Association, 2013), the diagnostic criteria for both AN and BN were widened to include presentations that, in the DSM-IV-TR (American Psychiatric Association, 2000), would have been considered EDNOS. Thus, of the 184 patients who fell in the EDNOS category, it is not clear who of them had purging behaviors and/or who might have better fit in an AN or BN category according to the DSM-5. Additionally, as there was no formal diagnosis for binge eating disorder (BED) or avoidant/restrictive food intake disorder (ARFID) in DSM-IV-TR, patients who would have met diagnostic criteria for these diagnoses were also diagnosed with EDNOS; therefore, patients in the EDNOS category were likely a heterogeneous group.

Also in Table 1, the mean length of stay in days for the four diagnoses followed a similar pattern for both adolescents and adults, with patients diagnosed with AN receiving the most treatment days, followed by patients diagnosed with BN and EDNOS, respectively. Patients diagnosed with EDNOS, whether adolescent or adult, received at least 16 fewer treatment days than patients diagnosed with AN-R, on average. However, on average, adolescents with purging behaviors—AN-P and BN—received 7.5 and 9.5 treatment days more than adults with purging behaviors, and adolescents diagnosed with EDNOS and AN-R received approximately 11 and 14 days more than adults diagnosed with EDNOS and AN-R, respectively. Mean length of stay for all adolescents was 47.7 days (*SD* = 15.1) and for all adults was 35.3 days (*SD* = 24.9); thus, treatment stays were, on average, 12 days longer for adolescents than adults.

### Status of the Total Samples of Adolescent and Adult Patients on Admission and Discharge

Both adolescents and adults, across ED diagnoses, were admitted to residential treatment with similarly impaired presentations of global functioning—that is, had average GAF scores in the mid-30s (indicating “major impairment”) with a range of 10–61 (see Tables 2, 3). Upon discharge, average GAF scores had risen into the low- to mid-40s (indicating “serious symptoms”) for adults and adolescents, respectively, with a range 10–70.

**TABLE 2:** Status on admission and discharge of adolescent ED residential patients by diagnosis.

	**Admission**			**Discharge**		
**Diagnosis**	***n***	***M***	**(*SD*)**	***n***	***M***	**(*SD*)**
**AN-R**						
GAF	377	35.6	(9.5)	372	47.8	(10.9)
Purge/last 30 days	375	3.6	(32.3)	312	0.0	(0.5)
Weight (lb)	398	94.3	(15.5)	392	107.4	(15.2)
%TBW	394	83.0	(9.0)	388	93.9	(7.2)
**AN-P**						
GAF	139	34.6	(9.5)	138	47.7	(11.4)
Purge/last 30 days	141	73.2	(188.8)	117	0.8	(5.7)
Weight (lb)	146	102.9	(15.0)	143	113.3	(14.0)
%TBW	145	86.5	(9.1)	142	95.3	(7.6)
**BN**						
GAF	294	33.5	(8.3)	299	46.7	(10.3)
Purge/last 30 days	304	112.0	(211.1)	211	0.5	(2.7)
Weight (lb)	306	133.8	(24.7)	304	135.6	(22.7)
%TBW	306	108.3	(17.4)	305	109.7	(15.9)
**EDNOS**						
GAF	124	37.4	(8.1)	125	45.6	(10.0)
Purge/last 30 days	123	13.2	(29.3)	68	0.1	(1.0)
Weight (lb)	128	130.1	(45.3)	128	133.5	(40.8)
%TBW	127	107.5	(30.8)	127	110.3	(26.0)

**Note*. Of the 979 adolescent patients, the 399 patients diagnosed with AN-R had a mean age of 14.6 (*SD* = 1.7) and mean length of stay (LOS) of 55.9 (*SD* = 30.6), the 146 patients diagnosed with AN-P had a mean age of 15.4 (*SD* = 1.5) and mean LOS of 50.1 (*SD* = 25.9), the 306 patients diagnosed with BN had a mean age of 15.8 (*SD* = 1.2) and mean LOS of 40.6 (*SD* = 25.1), and the 128 patients diagnosed with EDNOS had a mean age of 15.1 (*SD* = 1.6) and mean LOS of 36.7 (*SD* = 26.5).*

**TABLE 3:** Status on admission and discharge of adult ED residential patients by diagnosis.

	**Admission**			**Discharge**		
**Diagnosis**	***n***	***M***	**(*SD*)**	***n***	***M***	**(*SD*)**
**AN-R**						
GAF	115	35.4	(8.6)	117	44.1	(11.0)
Purge/last 30 days	114	7.8	(57.9)	71	0.0	(0.0)
Weight (lb)	120	100.4	(16.6)	119	110.4	(17.2)
%TBW	120	79.6	(8.7)	119	87.2	(9.0)
**AN-P**						
GAF	76	35.1	(6.4)	77	42.3	(8.9)
Purge/last 30 days	69	104.8	(204.1)	41	1.2	(4.9)
Weight (lb)	78	100.7	(14.2)	77	111.0	(14.2)
%TBW	78	81.1	(8.1)	76	89.4	(7.9)
**BN**						
GAF	185	34.9	(6.7)	181	42.7	(10.1)
Purge/last 30 days	181	93.2	(116.5)	87	1.3	(5.1)
Weight (lb)	188	135.8	(29.7)	186	136.6	(26.8)
%TBW	186	106.7	(20.7)	185	107.2	(18.0)
**EDNOS**						
GAF	55	37.4	(7.9)	53	45.2	(9.1)
Purge/last 30 days	51	85.2	(420.8)	19	1.6	(6.9)
Weight (lb)	56	165.4	(79.1)	56	164.4	(75.8)
%TBW	56	126.0	(49.8)	55	123.3	(45.5)

**Note*. Of the 442 adult patients, the 120 patients diagnosed with AN-R had a mean age of 25.4 (*SD* = 10.3) and mean length of stay (LOS) of 41.7 (*SD* = 25.7), the 78 patients diagnosed with AN-P had a mean age of 24.7 (*SD* = 7.6) and mean LOS of 42.5 (*SD* = 30.1), the 188 patients diagnosed with BN had a mean age of 23.3 (*SD* = 6.9) and mean LOS of 31.1 (*SD* = 20.0), and the 56 patients diagnosed with EDNOS had a mean age of 29.6 (*SD* = 13.4) and mean LOS of 25.4 (*SD* = 18.0).*

Whereas the patients diagnosed with AN-R, unsurprisingly, had relatively few purge episodes on admission (calculated per 30 days in Tables 2, 3; equivalent to a mean of 0.8 and 1.8 per week for adolescent and adult patients, respectively), the adolescent and adult patients diagnosed with AN-P reported purging equivalent to a mean of 17.1 and 24.5 times per week, respectively, and the adolescent and adult patients diagnosed with BN reported purging equivalent to a mean of 26.1 and 21.7 times per week, respectively. Interestingly, adolescent and adult patients diagnosed with EDNOS reported purging equivalent to a mean of 3.1 and 19.9 times per week, respectively, which means that some of the patients, according to the DSM-IV-TR (American Psychiatric Association, 2000) diagnostic criterion for purge frequency alone, would qualify for a diagnosis of BN; however, as these patients were given a diagnosis of EDNOS instead of BN, it is likely that these patients did not have binge behaviors or other diagnostic criteria required for BN. On discharge, mean scores for patients in all diagnostic categories indicated that virtually no purge behavior had been evident/reported in the 30 days prior to discharge. Of all patients who reported having purge behavior the month before admission and had purge data at discharge (*n* = 726), 89.1% were able to completely cease purging while in treatment.

On admission, adolescents diagnosed with AN-R and AN-P had a mean weight that represented 83.0 and 86.5% of minimum TBW, respectively; on discharge, mean %TBW for adolescent patients diagnosed with AN-R and AN-P was 93.9 and 95.3, and the difference in mean weight was 13.1 and 10.4 lb, respectively (see Table 2). For adults diagnosed with AN-R and AN-P, mean %TBW on admission was 79.6 and 81.1, respectively; whereas, on discharge, mean %TBW for adults diagnosed with AN-R and AN-P was 87.2 and 89.4 (see Table 3), and the difference in mean weight was 10.0 and 10.3 lb, respectively. Although it was clear that the weight status of patients diagnosed with AN was closer to the minimum TBW on discharge than on admission, because diagnostic criteria for the diagnosis of AN per DSM-IV-TR (American Psychiatric Association, 2000) included a %TBW of less than 85, it was also clear that many patients in the sample diagnosed with AN (40.1%) had been partially (85% TBW or above) or even fully weight restored prior to being admitted for residential treatment. (Although not typical, it is also not uncommon for patients to weight restore in a hospital setting before stepping down the continuum of care to residential treatment.) Therefore, to get a more accurate portrayal of weight restoration, it was important to analyze the subset of the patients who needed to gain weight at the time of admission (results are discussed below). Additionally, as weight was measured only once per week, for patients’ weight change to be captured reliably, they must have had a length of stay of at least 7 days to capture weight change.

### Weight Restoration for the Subset of AN Patients

For the smaller sample (*n* = 411) of adults and adolescents diagnosed with AN who were admitted with weight that was below 85% of their minimum TBW and who remained in treatment for 7 or more days (see Table 4), mean %TBW was in the mid to high 70s, with adolescents being admitted with a slightly higher mean %TBW than adults. Adolescents were also discharged at a higher %TBW (at a mean of 91.7 and 90.8 for AN-R and AN-P, respectively) than adults (84.7 and 87.7 mean %TBW for AN-R and AN-P, respectively). Whereas adolescents diagnosed with AN-R and AN-P gained 17.1 and 15.1 lb, representing an increase in %TBW of 14.6 and 12.3, respectively, adult patients diagnosed with AN-R and AN-P gained 12.3 and 12.5 lb, representing an increase in %TBW of 9.5 and 10.4. Although adolescents made larger gains than adult patients, it is important to note that adolescents had longer lengths of treatment than adults. Analyses on rate of weight change (i.e., the mean of patients’ weight change divided by days of treatment multiplied by 7 days in a week) indicated that adolescents with active AN-R and AN-P had a mean rate of weight increase of 2.0 and 1.8 lb per week, respectively, whereas adults with active AN-R and AN-P had a rate of weight increase of 2.1 and 2.0 lb per week, respectively. Thus, rate of weight restoration was similar, with adults gaining slightly faster. Adult patients gained an average of 2 BMI points, going from a mean BMI of 15.7 to 17.7 for AN-R and from 16.2 to 18.2 for AN-P. Although, as discussed above, BMI is not standardized across adolescents and is therefore not presented in the table, it may be noteworthy that adolescents diagnosed with AN-R and AN-P gained an average of 2.9 and 2.5 BMI points, respectively, from admission to discharge.

**TABLE 4:** Change in weight of adolescent and adult patients with active AN on admission.

	**Admission**		**Discharge**		**Difference**	
	***M***	**(*SD*)**	***M***	**(*SD*)**	***M***	**(*SD*)**
**Adolescent**						
AN-R (*n* = 225)^a^						
Weight (lb)	89.5	(12.9)	106.6	(14.2)	17.1	(7.9)
%TBW	77.1	(5.0)	91.7	(5.9)	14.6	(6.4)
AN-P (*n* = 60)^b^						
Weight (lb)	92.9	(11.2)	108.0	(12.9)	15.1	(8.3)
%TBW	78.5	(4.7)	90.8	(6.4)	12.3	(6.5)
**Adult**						
AN-R (*n* = 74)^c^						
Weight (lb)	93.6	(12.4)	105.8	(14.7)	12.3	(8.3)
%TBW	75.2	(6.2)	84.7	(7.4)	9.5	(6.2)
BMI	15.7	(1.4)	17.7	(1.6)	2.0	(1.3)
AN-P (*n* = 52)^d^						
Weight (lb)	96.7	(13.4)	109.2	(14.1)	12.5	(7.8)
%TBW	77.3	(6.0)	87.7	(7.6)	10.4	(6.7)
BMI	16.2	(1.2)	18.2	(1.5)	2.0	(1.4)

**Note*. Active AN was defined as having been diagnosed with either AN-R or AN-P and having a weight below 85% TBW at admission. As weight was measured once per week in treatment, the analyses of weight change only included data from patients who were in treatment 7 or more days (data from 12 adolescent patients and 10 adult patients were excluded due to having less than 7 treatment days). Additionally, the weight analyses only included patients who had data for both admission and discharge weight and %TBW (six additional adolescent patients and one additional adult patient were missing these data so were excluded). ^*a*^The 225 adolescent patients diagnosed with AN-R included here had a mean length of stay (LOS) of 61.4 (*SD* = 28.0). ^*b*^The 60 adolescent patients diagnosed with AN-P included here had a mean LOS of 57.1 (*SD* = 26.0). ^*c*^The 74 adult patients diagnosed with AN-R included here had a mean LOS of 44.2 (*SD* = 23.3). ^*d*^The 52 adult patients diagnosed with AN-P included here had a mean LOS of 46.3 (*SD* = 32.3).*

## Discussion

This paper summarizes the data available on admission and discharge for 1,421 patients—96% of all patients who were treated for an ED between the years 2006 and 2013 at a large, multi-location residential treatment program. Results suggest that, (1) for patients who needed weight restoration upon admission, adolescent and adult patients gained 2.0 and 2.1 lb/week, respectively; (2) of patients who reported purge behavior the month before admission, 89.1% were able to completely cease purging while in treatment; (3) although improvement of approximately 10 mean GAF points was made during treatment, patients were still quite impaired at discharge; and (4) mean length of stay was 12 days longer for adolescents than adults, and 10–15 days longer for patients diagnosed with anorexia compared to bulimia or EDNOS, respectively.

Prior to this paper, there have been eight studies in the literature that have reported discharge outcomes on a total of 1,157 patients treated for EDs in residential facilities. Those 1,157 patients represented less than 65% of the patients treated during those studies (potentially much less, as some studies had small, non-random, convenience samples). The addition of the 1,421 patients in the current study more than doubles the total number of patients with outcomes treated in ED residential facilities reported in the literature. Furthermore, because this study includes data on 96% of patients treated during the study period, it represents a significant increase from the aggregate percentage of data available in previous studies.

The clear majority (96.9%) of the patients in this study had received ED treatment prior to their admission to Center For Discovery—over 80% had prior outpatient and almost half had prior hospitalization. Such a high percentage of patients having past treatment is typical for this level-of-care (Brewerton and Costin, 2011). As prior research suggests that most EDs start during adolescence (Micali et al., 2013), it is unsurprising that the percentages of adults who had received treatment in one or more of the middle levels-of-care on the continuum (i.e., intensive outpatient, partial hospitalization, or non-Discovery residential) were as much as double the percentage of adolescents, as adult patients likely had experienced a longer duration of illness and therefore more opportunity for treatment.

Most patients in this study were diagnosed with AN, followed by BN, and then EDNOS. Examining the other residential outcome studies, AN is the most common of the EDs treated in residential care. However, some studies showed a higher prevalence of patients diagnosed with EDNOS than BN (Weltzin et al., 2014). The findings that AN is the most commonly treated ED diagnosis in residential care may be due to the nature of insurance coverage and the requirement for “medical necessity,” as weight suppression is one of the easier metrics of medical necessity to quantify. In line with this reasoning, patients diagnosed with AN received longer treatment lengths than patients diagnosed with other EDs—10–15 days more for AN compared to BN, and 15 + more days for AN compared to EDNOS. The length of stay difference between AN and BN is reflected in the literature base (e.g., Delinsky et al., 2010; Brewerton and Costin, 2011); however; some studies show different patterns between the length of stay for BN and EDNOS, with patients diagnosed with EDNOS receiving up to 10 more days of treatment than patients diagnosed with BN (Delinsky et al., 2010).

Although GAF scores improved over the course of treatment, the means increased from the mid-30s—“major impairment”—on admission to the mid-40s—“serious symptoms”—on discharge, with adolescents scoring slightly higher (showing less impairment) than adults on discharge. GAF scores have not been retained in the DSM-5 because of a “conceptual lack of clarity… and questionable psychometrics in routine practice” (American Psychiatric Association, 2013, p. 16). However, it may be important to note that: (a) GAF scores did not vary predictably by ED diagnosis, (b) functioning was very impaired at admission, and (c) although noticeable and reliable improvement (approximately 10 points) was made over the course of treatment, patients were still quite impaired at discharge, supporting the need for a continuum of care, especially partial hospitalization and intensive outpatient programs.

Among the patients with purging behaviors on admission, the patients diagnosed with BN (*n* = 456) reported purging equivalent to a mean of 3.7 times per day on admission and the patients diagnosed with AN-P (*n* = 184) reported equivalent to a mean of 3.2 times per day. Both groups reported virtually no purging at time of discharge (on average, less than 0.03 per day). Looking at all patients who had purging behavior on admission and purge data on discharge (*n* = 726), almost 90% were able to completely cease purging while in treatment.

The patients treated for AN (*n* = 686) had a mean increase in BMI of 2.1 and a mean length of stay of 53.7 days. Examining just the patients diagnosed with AN who met DSM-IV-TR (American Psychiatric Association, 2000) diagnostic criteria (*n* = 411), the mean BMI increase was 2.6 points and the mean length of stay was 55.8 days. This increase is higher than findings reported by the other residential ED treatment programs included in Friedman et al.’s (2016) review [i.e., an increase of 2.3 in Brewerton and Costin (2011); of 1.7 in Lowe et al. (2003); of 2.0 in McHugh (2007); and of 1.9 in Weltzin et al. (2014)]. However, the mean length of stay of 55.8 days in this study, is less than some of the mean lengths of stay reported by the other studies—for example, Brewerton and Costin (2011) study with a mean length of stay of 98 days—and greater than the mean length of stay of other studies.

The change in BMI in the patients diagnosed with AN represents a mean weight gain of approximately 12.4 lb (1.7 lb per week) and an increase of approximately 10% TBW, from 82.8 to 93.0% TBW. For the subset of these patients who met DSM-IV-TR diagnostic criteria on admission (American Psychiatric Association, 2000), the BMI change represents a mean weight gain of approximately 15.3 lb (2.0 lb per week) and an increase of approximately 12% TBW, from 77.0 to 89.8%TBW. By way of comparison, in a study of patients treated in 11 adolescent-medicine, outpatient ED programs, only one half of patients achieved a weight gain to 90% TBW (IBW) over a *1-year* treatment period (Forman et al., 2011). Thus, although almost two months of ED treatment in a residential facility can be expensive (albeit, less expensive than at an inpatient hospital setting), weight restoration in residential treatment is achieved at a more rapid pace. Such timely weight restoration may be vital—ED researchers cite slow and low weight restoration as dangerous, as it results in not just the eventual risk of bone disease and relapse, but also a decline in motivation for recovery (Steinhausen, 2002; Strober and Johnson, 2012). And although there is no way to make a direct comparison, it is known clinically and reinforced in these data that most patients referred for residential treatment have not been successful in outpatient treatment settings.

Adolescent and adult patients varied in almost all domains examined: prior treatment, most prevalent diagnosis, length of treatment received, and symptom improvement. Higher percentages of adults had received levels-of-care that fall in the middle of the continuum; whereas, a higher percentage of adolescents had been hospitalized previously. Adolescents were more often diagnosed with AN, whereas adults were more often diagnosed with BN. Adolescents received more days of treatment than adults—12.5 days more on average; this may be because the adults were less willing to stay as long and/or because insurance companies authorized shorter treatment lengths for adults. Adolescents had more favorable outcomes (though some only slightly more favorable) than adult patients in terms of GAF scores, purging frequency, and weight gain. Notably, adolescents, but not adults, diagnosed with AN were able to weight restore to above an average of 90% TBW. Reaching the benchmark of 90% TBW, particularly for developing adolescents, is important for a number of reasons including: (a) at 90% TBW there is often a marked reduction of symptoms of malnutrition (Strober et al., 1997); (b) evidence that psychopathological symptoms can persist for years when weight restoration is incomplete (Strober and Johnson, 2012); and (c) findings that show a stable relationship between achieving at least 90% TBW at discharge and long-term weight maintenance (Couturier and Lock, 2006). However, this difference in improvement between adolescent and adult patients must be examined within the context of adolescents receiving 34% more time in treatment than adults. In fact, the rate of weight restoration was actually slightly higher for adults than for adolescents—2.1 and 2.0 lb per week, respectively. This finding suggests that if adults were provided lengths of treatment similar to adolescents, adults would also achieve 90% TBW and strengthening their chance for long-term weight maintenance and lasting recovery.

## Conclusion

In summary, the data in this study answer the call presented by Attia et al. (2016) by demonstrating the outcomes for almost 1,500 patients treated in residential care, showing that adolescent and adult patients, across ED diagnoses, made clinically significant improvements, with longer lengths of stay possibly leading to greater improvements. Strengths include sample size and participation rate, especially given the lack of available data in the field. Limitations of this study include the high variance of some variables (e.g., reporting purging with means and standard deviations may not fully reflect the skewed distribution of this variable); reliance on self-report and parent-report for admission data; the omission of binge data in the collection process; and, due to the diagnostic standards of the time, the categorizing of patients who would today be diagnosed with BED or ARFID into the diagnosis of EDNOS. Additionally, as this study was based in the United States and examined the US structure of ED residential treatment, results may not be generalizable to different levels-of-care available in other countries. Future research could examine reason for discharge to explore why adult patients do not receive the treatment lengths that adolescents do, and could control for variables like insurance and prior treatment history. Lastly, an important next step will be to study whether the accomplishments achieved by the patients over the course of treatment are maintained; such studies are underway.

## Data Availability Statement

The dataset generated for this study is available for review on request to the corresponding author.

## Ethics Statement

This study was reviewed and approved by the IRB of Northwell Health. Written informed consent to participate in this study was provided by the participants’ legal guardian/next of kin.

## Author Contributions

MF oversaw the analysis and writing. JH and SC were responsible for research design, oversaw data collection and de-identifying, and participated in the writing. RF, VF, LM, and JM supported the analysis, participated in the interpretation, and reviewed the manuscript. MS and SL contributed to data collection and reviewed the manuscript. JF cleaned and coded the data and ran analyses.

## Conflict of Interest

JH, SC, RF, MS, and SL were or are employed by Center For Discovery.

The remaining authors declare that the research was conducted in the absence of any commercial or financial relationships that could be construed as a potential conflict of interest.
